# Shitsan Pai: the establishment of the first biophysics department in the world

**DOI:** 10.1007/s13238-018-0557-0

**Published:** 2018-06-11

**Authors:** Rui Liu

**Affiliations:** Department for the History of Science and Scientific Archaeology, University of Science and Technology of China, Hefei, 230026 China

Since the birth of New China, it suffered from many internal and external problems, and thousands of things waited to be done (Fig. 1). In order to improve its status in the international community, and consolidate its national defense, the Chinese government decided to develop nuclear weapons and aerospace industry. In 1954, the Soviet Union leader Khrushchev had an official visit to China. During his visit, Chairman Mao Zedong put forward a request to Moscow to help China manufacture nuclear weapons. But Khrushchev did not agree to this request. He gave the reason that China does not yet have the corresponding industrial base and economic strength. In the absence of support of the Soviet Union at that time, the Chinese government carried out the project by relying on its own scientific research teams. And this is a long-term project. We must not only need to develop nuclear weapons, but also understand the damage to human body and organism of the nuclear weapons, and find ways to prevent the injuries. Therefore, the establishment of the corresponding disciplines to conduct a systematic study is also conforming to the national macro-level consideration.

**Figure 1:**
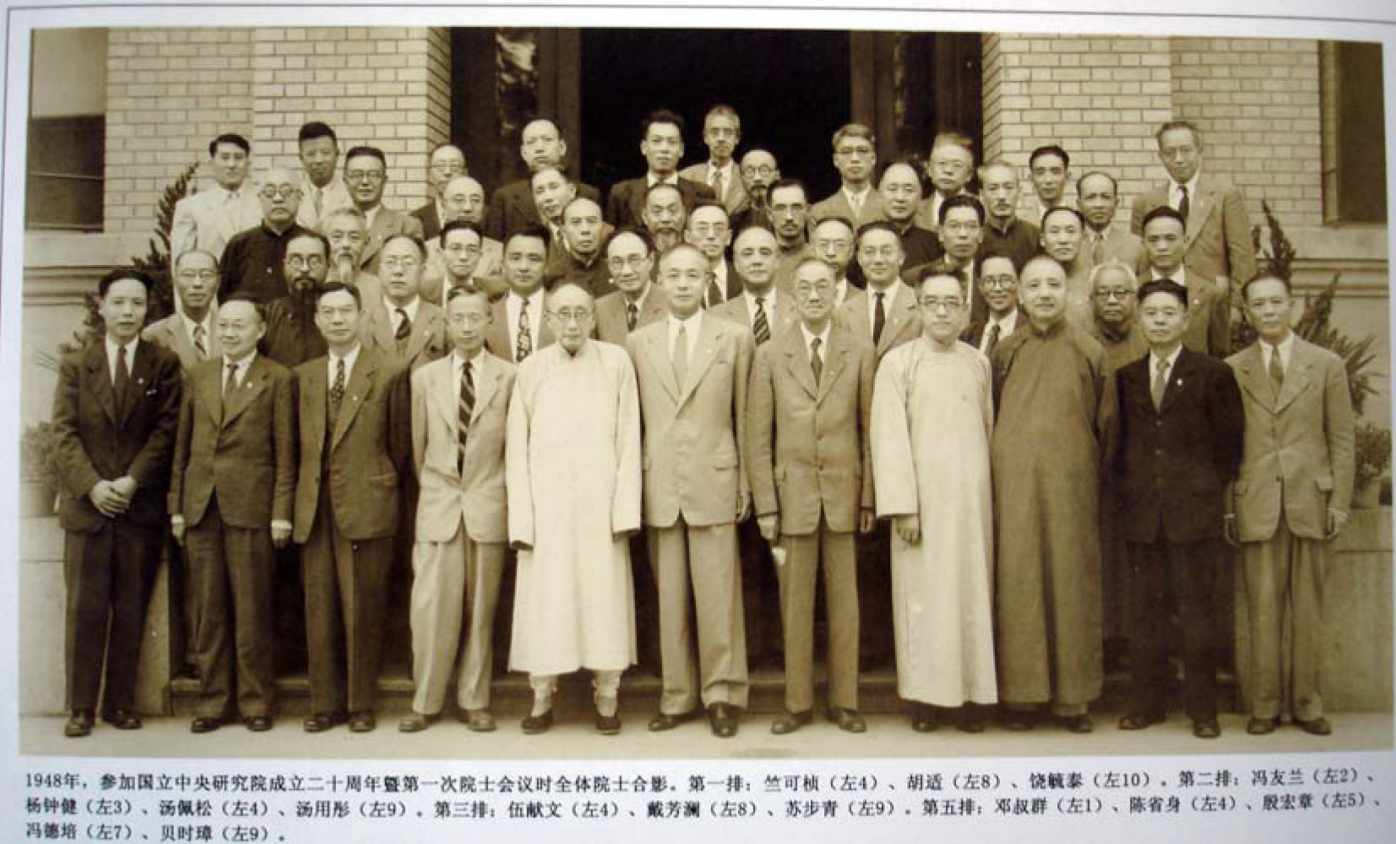
In 1948, the first academician conference of the national central research institute, the ninth from left of the fifth row is Shitsan Pai

At the same time, another important project, the aerospace project, was in the intensive process. The original intention was to serve the military. In 1950s, New China was still under the blockade, siege and threat of imperialism. In order to defend our land, the top leadership of the Central Committee decided to develop our own missile cause. In October of 1956, China’s first special missile (rocket) research institute, the Ministry of Defense 5th Research Institute was formally established. Since then, China’s aerospace industry started moving on a fast pace. In November 16th of 1957, the second branch of the 5th Institute of National Defense, a research institute which specializing in missile control systems, was formally established, which is the predecessor of the second Academy of Aerospace Science and Technology of China. Premier Zhou Enlai appointed lieutenant general Zheng Wang as president of the second branch (Chrisluk, 2015). With the development of aerospace industry, manned space flight has also been researched. Thus, it is urgent to study the influence of zero gravity on organism in biophysics.

In 1958, China launched experimental rockets to pave the way for military missile and space research. In 1958, China began to develop sounding rockets. The T-7 liquid sounding rocket and the improved T-7A sounding rocket were developed successively, and in 1965, the solid sounding rockets “Peace 2” and “Peace 6” were developed. “The implementation of the T-7A biological sounding rocket highlights the dialectical form of interaction between biophysicists and states. The discipline of biophysics is embedded in the general historical context in which science is used to serve the interests of the nation” (Chrisluk, 2015).

In 1950s, Biophysics as an independent discipline was born. It made Shitsan Pai (Fig. 2) realized that biology could make great progress and finally reveal the nature of life only through further research in biophysics and biochemistry. For this purpose, during his three-month visit to the Soviet Union in 1956, Shitsan Pai focused on the research of radiobiology, biophysics and biochemistry of the USSR Academy of Sciences and a number of institutions of colleges and universities in USSR. With great foresight and courage, Shitsan Pai undertook the task of establishing the Institute of Biophysics of the Chinese Academy of Sciences after prepared carefully. In July 1958, Shitsan Pai proposed to the Chinese Academy of Sciences to change the name of the Laboratory Biophysics Institute to the Institute of Biophysics. In July 29th 1958, the 9th executive meeting of the Chinese Academy of Sciences approved the plan of transforming the Beijing Institute of Experimental Biology into the Institute of Biophysics, which was formally approved by the State Council in September 26th, 1958. And Shitsan Pai was also appointed the first director of the Institute of Biophysics. The Institute of Biophysics was one of the few specialized biophysical research institutes in the world at that time, its establishment marks the biophysics as an independent discipline was established in China and laid a solid foundation for its vigorous development (Wang, 2010).

**Figure 2:**
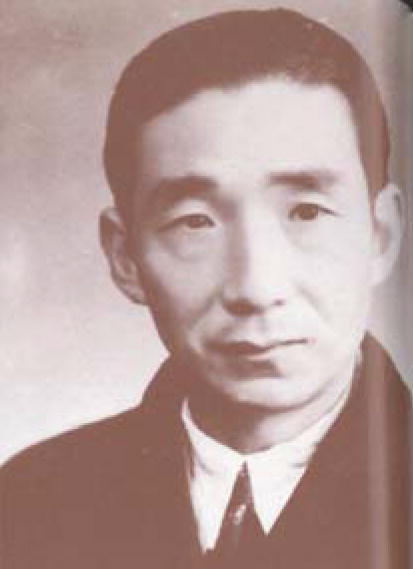
Middle age of Shitsan Pai (1953)

Shitsan Pai (Fig. 3), as the first director of the Institute of Biophysics, Chinese Academy of Sciences, has learned some classical biology abroad, such as observing cells, embryo development and so on. The relationship with mathematics, physics and chemistry is relatively weak. With the rapid development of mathematics, physics and chemistry in the 1940s and 1950s, Shitsan Pai believed that biology must be combined with these disciplines. Biologists must have a solid mathematical and physical foundation so that they can keep up with the times.

**Figure 3:**
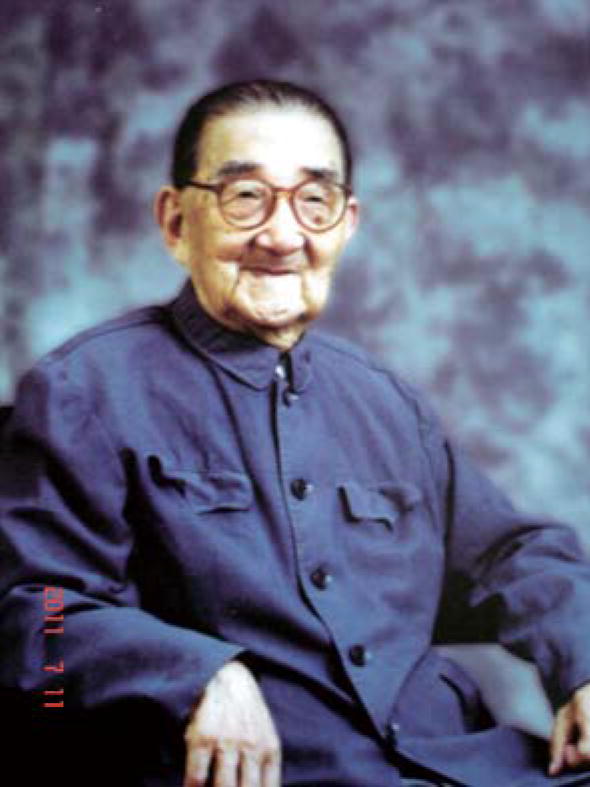
Shitsan Pai (1903–2009)

By using modern and emerging physical theories and techniques in quantitatively analyzing biological phenomena, we have raised our research on previous organisms to a new stage. At the time, China is developing nuclear weapons and exploring aerospace. The establishment of the Institute of Biophysics plays a very important role in these two studies.

At the same time, in order to cultivate its own scientific and technological talents, the Chinese Academy of Sciences decided to establish its own university. In February 13th 1958, the meeting of directors from research institutes of the Academy of Sciences was held in Beijing. The meeting put forward the slogan “Scientific work must leap forward”. Moruo Guo who was the President of the Chinese Academy of Sciences pointed out this call at the meeting which was in response to the CPC Central Committee’s call to “march toward science” and “strive to approach and catch up with the world’s advanced level in certain badly needed and important scientific departments within 12 years”. Some research institutes have put forward their own views on the courses to train the talents they need. One stone has stirred up a thousand waves, and many directors have also expressed their desire to train their own talents. Therefore, under the situation of the “Great Leap forward in Education” at that time, the Academy of Sciences decided to set up its own university (1958).

Shitsan Pai played an important role in the establishment of the Department of Biophysics at the University of Science and Technology of China (USTC). In June 1958, USTC set up a Preparatory Committee to plan the establishment of 13 departments systematically, and they have solicited the opinions of the directors of each institute. Shitsan Pai suggested that a test point should be carried out in USTC. The establishment of the Department of Biophysics in USTC can provide talents for the Institute of Biophysics in order to the following research.

The personal influence of Shitsan Pai has played an important role in the establishment of the Department of Biophysics. Firstly, he advocated of establishing Department of Biophysics in USTC. Because he believed that researchers should keep pace with the times. Secondly, he thought that we should train more people for the following research of biophysics for our country. Thirdly, he considered that if we still set up specialties in traditional biology, such as Botany, Zoology, Microbiology and other specialties, we would not have any advantages, and cannot achieve the goal that the study must relate to the current national macro strategy (Fig. 4). Thus, when meeting with the problem of specialty setting under the Department of Biophysics, Shitsan Pai thought that we can only set Biophysics temporarily. This was consistent with the national defense scientific research task called “Two bombs and one Satellite” at that time. Soon, the Preparatory Committee agreed this proposal. As the representative of Biology, the Department of Biophysics became one of the 13 departments started firstly.

**Figure 4:**
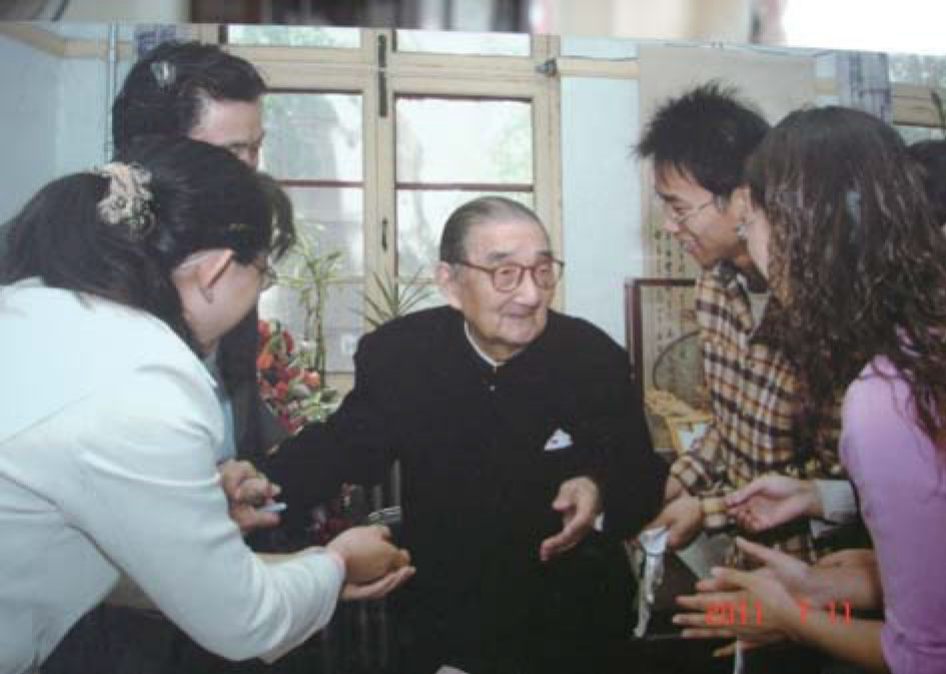
Shitsan Pai and the visiting students

One of Shitsan Pai’s important study ideals is: “Scientific research should serve the national construction”. After the establishment of the Institute of Biophysics, not only in basic research, but also in connection with national defense, industry, agriculture, medicine and biotechnology, it can make important contributions to the country.

The students at that time had a high opinion of Mr. Shitsan Pai. For example, Yunyu Shi^1^, first dean of the College of Life Sciences in USTC, was a 1960 class student in the Department of Biophysics. And she was chosen as academician of Chinese Academy of Sciences in 1997. In her memory, she has mentioned Shitsan Pai’s school-running idea: “*Aim at the front of the subject*, *the biology departments from other colleges and universities had done a lot of work in traditional biology study at that time*. *If USTC re-establish a biology department like this, there would not be many new ideas*, *and it would be very difficult to create new research directions*. *Mr*. *Pai led us to establish the Department of Biophysics*, *which is aimed at the frontier*. *He believed that the development of science is changing quickly with each passing day*, *so is biology*. *And the development of science has always been based on mathematics*, *physics and other disciplines*. *Students should lay a solid physical and chemical foundation in school*, *so no matter how science develops and changes in the future*, *they were able to integrate quickly* (Xiong, 2017).”

As the student representative of 1959 class, Department of Biophysics in USTC, Professor Tiande Shou^2^ also has a very high appraisal to Shitsan Pai: “*He thought that the classical biology he learned in Europe*, *such as microscopic observations of cell or embryonic development, which was advanced in the 1920s and 1930s*. *By 1940s and 50s*, *mathematics*, *physics and chemistry had infiltrated*. *The requirements for scientists are much higher than in the past*. *So he was going to set up a biophysical institute*. *He thought that scientists who will do biological work in the future should be good at mathematics*, *physics and advanced experimental technology*. *This kind of discipline must be studied by the people who have strong interdisciplinary abilities*. *This is a view from a strategic height*. *So he thinks that the establishment of the Department of Biology at the Chinese University of Science and Technology is about to establish the Department of Biophysics*” (Liu, 2017).

The Department of Biophysics in USTC is not only in China, but also the only biophysical “department” in the world at that time. The colleges and universities in other countries had only set up biophysics courses or establish biophysics research direction. Establish a department of biophysics, and students are trained in biophysics course systematically, which was the first time ever.

The initial positioning of the Department of Biophysics which guided by Shitsan Pai was: “The graduate students from Department of Biophysics should possess the general biophysical theory and some experimental techniques. After practice for a certain period of time, they could undertake scientific research in the field of biophysics, or a teaching job in the college, and could also serve as an information staff in scientific research” (Fig. 5). Biophysics was a new and sophisticated science at the time, which was also a frontal subject. This subject contains so much, and could be divided into the following four directions: the first is to grasp the physical basic structure, performance and movement clearly; the second is to understand the energy metabolism of organisms; the third is the information and control problems of organisms; the fourth is skilled in using biophysical instruments. Thus, the contents of the main courses in Department of Biophysics include the following four aspects: Radiobiology, Isotope application in biology and Dosimetry, Biophysics, Biophysical instruments and technology.

**Figure 5:**
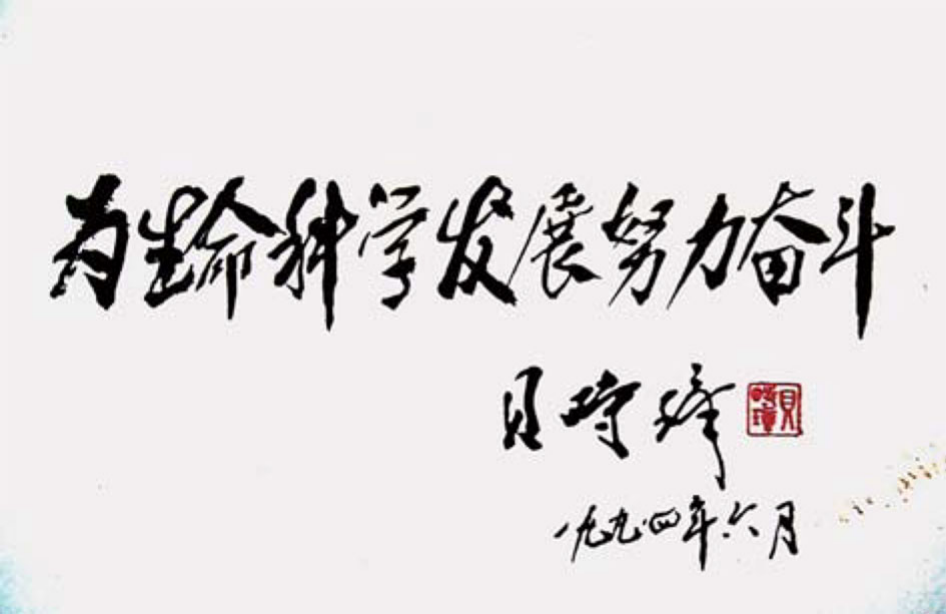
Shitsan Pai’s inscription in 1994

From the view of the specialty setting of the Department of Biophysics, we can clearly see that the cultivation goal of the Department of Biophysics is obviously different from that of the traditional specialties in biology. There were no animal biology, plant biology, microbiology, genetics and so on. And they were replaced by the specialties which related to radiation control and space exploration. This measure served to the “Two bombs and one Satellite” project very well. It also reflects the purpose of science and technology should serve for the political and military.

In order to let the first batch of students become familiar with the contents of their specialty as soon as possible, Shitsan Pai taught the students by himself in the first few years of the establishment of the department. And a few years later, he handed over this work to the deputy director of the department, Shumin Shen, who was a researcher from the Institute of Biophysics. The first three classes of the Department of Biophysics in USTC, from 1958 to 1960, had more than 200 graduates. They became the backbone of the early development of biophysics in China.

## Footnotes

1 Yunyu Shi, female, was born in April 21st 1942 in Chongqing. She is currently a professor at the University of Science and Technology of China. In 1997, she was elected as an academician of the Chinese Academy of Sciences. In 2009 she was elected as an academician of the third World Academy of Sciences. In 1965, she graduated from the Department of Physics, University of Science and Technology of China, majoring in Biophysics. Since 1970, she has been an assistant professor, lecturer, associate professor and professor at the University of Science and Technology of China.

2 Tiande Shou, male, Professor, doctoral supervisor, was born in 1941in Xi’an. He graduated from the Department of Biophysics at USTC in 1964, and then he stayed and taught at USTC. He served as a deputy director, director of the Department of Biology and associate dean of College of Science.
